# Prospective study on breastfeeding, lipid profile and cardiovascular risk markers in women with familial hypercholesterolaemia: study protocol for the FH-FEMINA study

**DOI:** 10.1136/bmjopen-2024-092208

**Published:** 2025-04-27

**Authors:** Marianne Klevmoen, Janneke W C M Mulder, Martin P Bogsrud, Kjetil Retterstøl, Elisabeth K Vesterbekkmo, Eva K R Pedersen, Tomas Freiberger, Michal Vrablik, Martina Vaclova, Anders Hovland, Nils Tore Vethe, Hilde Kristin Brekke, Per Ole Iversen, Marit Veierød, Jeanine Roeters van Lennep, Kirsten B Holven

**Affiliations:** 1 Norwegian National Advisory Unit on Familial Hypercholesterolemia, Oslo University Hospital, Oslo, Norway; 2 Department of Nutrition, Institute of Basic Medical Sciences, University of Oslo, Oslo, Norway; 3 Department of Internal Medicine, Erasmus MC Cardiovascular Institute, University Medical Center Rotterdam, Rotterdam, The Netherlands; 4 Unit for Cardiac and Cardiovascular Genetics, Oslo University Hospital, Oslo, Norway; 5 The Lipid Clinic, Oslo University Hospital, Oslo, Norway; 6 Clinic of Cardiology, St. Olavs University Hospital, Trondheim, Norway; 7 Department of Heart Disease, Haukeland University Hospital, Bergen, Norway; 8 Department of Clinical Science, University of Bergen, Bergen, Norway; 9 Centre of Cardiovascular Surgery and Transplantation, Brno, Czech Republic; 10 Medical Faculty, Masaryk University, Brno, Czech Republic; 11 National Institute for Metabolic and Cardiovascular Disease Research, Brno, Czech Republic; 12 Third Department of Internal Medicine, General University Hospital, First Faculty of Medicine, Charles University, Prague, Czech Republic; 13 Nordland Heart Center, Bodo, Norway; 14 Department of Pharmacology, Oslo University Hospital, Oslo, Norway; 15 Department of Pharmacy, University of Oslo, Oslo, Norway; 16 Department of Haematology, Oslo University Hospital, Oslo, Norway; 17 Oslo Centre of Biostatistics and Epidemiology, Department of Biostatistics, Institute of Basic Medical Sciences, University of Oslo, Oslo, Norway

**Keywords:** Coronary heart disease, Cardiovascular Disease, Maternal medicine, Postpartum Women, Primary Prevention

## Abstract

**Introduction:**

Early and lifelong treatment is essential in patients with familial hypercholesterolaemia (FH) due to genetically elevated low-density lipoprotein cholesterol (LDL-C) from the first years of life. In women with FH, lipid-lowering treatment is interrupted during childbearing years due to contraindication of the medication during conception, pregnancy and breastfeeding. However, little is known about the impact of breastfeeding on lipid profile and other risk markers for atherosclerotic cardiovascular disease (ASCVD) in women with FH compared with women without hypercholesterolaemia, and to what extent statins transfer into breast milk.

We aim to investigate (1) the association between breastfeeding and serum lipid profile in women with and without FH; (2) the association between breastfeeding and other ASCVD risk markers in women with and without FH and (3) the concentration of statins in breast milk of women with FH.

**Methods and analysis:**

FH-FEMINA is a prospective study aiming to include 50 women with FH in Norway, the Netherlands and the Czech Republic. Additionally, 20 women without hypercholesterolaemia will be enrolled as a control group in Norway. Women will be included at the first study visit in gestational week 36, and follow-up visits will be scheduled at 2–4 weeks, and at 3, 6, 9 and 12 months postpartum. Information on lifestyle factors, treatment history and current and previous pregnancies will be collected. At each visit, a non-fasting blood sample, breast milk sample and information on diet, body mass index and blood pressure will be collected. Additional blood samples will be collected from the women with FH at 2, 4, 5, 7, 8, 10 and 11 months postpartum for as long as they are breastfeeding. At (re-)initiation of statin treatment, breast milk samples from women with FH will be collected for drug concentration measurements.

**Ethics and dissemination:**

Ethical approval will be obtained prior to study start in all three countries. Participants will be informed about the study and receive ample time to ask questions before the informed consent form is signed. The findings from this study will be disseminated to healthcare professionals, researchers and patients via peer-reviewed scientific article(s), conferences, patient organisations and social media.

**Trial registration number:**

NCT05367310.

**STRENGTHS AND LIMITATIONS OF THIS STUDY**

- A strength of the study is the use of repeated measurements to enable close monitoring of the lipid level variations in women with and without familial hypercholesterolaemia (FH) during the first year postpartum.
- Another strength is the well-characterised FH diagnosis, which is genetically verified (for most of the participants) or clinically diagnosed.
- A limitation of the study is the small sample size, reflecting the limited availability of pregnant women with FH within the timeframe of this study.
- The absence of control group in two of the three countries is a limitation.
- Variability in breastfeeding practices (frequency, duration) is another limitation that may influence the lipid levels in the first year postpartum.

## Introduction

 Patients with familial hypercholesterolaemia (FH) have genetically elevated levels of low-density lipoprotein cholesterol (LDL-C) from the first years of life. The lifelong cholesterol burden throughout life defines the risk of atherosclerotic cardiovascular disease (ASCVD).^1 2^

In female patients with FH, lipid-lowering treatment is discontinued during childbearing years due to contraindication of the medication during conception, pregnancy and breastfeeding.^3^ Additionally, cholesterol levels increase as part of the normal physiological changes during pregnancy.^4 5^ This pregnancy-related relative increase in LDL-C is similar between women with and without FH, ranging from approximately 30% to 50%.^6^ However, the absolute LDL-C levels at the end of pregnancy are considerably higher in women with FH (mean 8.6 mmol/L) compared with women without FH (mean 3.9 mmol/L).^6^

In a previous study among 80 women with FH from Norway and the Netherlands, using a self-reported questionnaire, we observed a median total duration of pregnancy-related off-statin periods of 2.3 years, from the planning phase of the first pregnancy to the end of breastfeeding of the last child.^7^ However, there were large individual variations, with off-treatment periods ranging from 0 to 14 years. Women with a higher number of children had a longer total duration without lipid-lowering treatment, and 20% of the participants had pregnancy-related off-statin time of more than 4 years. Almost 90% of the women wished to receive more information from healthcare professionals on pregnancy and breastfeeding regarding FH, especially from their general practitioner (GP).^7^

Few, if any, studies on the effects of breastfeeding in women with FH exist. Studies in the general population show that breastfeeding has several benefits for both the mother and the child.^8^ Maternal benefits may include facilitating postpartum weight loss and improving lipid profile.^8 9^ There are also studies indicating that breastfeeding can reduce the risk of type 2 diabetes and cardiovascular disease in women who have breastfed.^8 10^

WHO recommends exclusive breastfeeding for the first 6 months, followed by continued partial breastfeeding up to 2 years of age or longer.^11^ Regarding FH, current guidelines for the management of dyslipidaemias do not provide specific recommendations regarding breastfeeding. Thus, women with FH are usually recommended to follow the same guidelines as those for women without FH.

LDL-C levels decrease postpartum; however, little is known about when and how rapidly the cholesterol levels decrease during breastfeeding in women with FH compared with women without FH. Moreover, the knowledge on the transfer of statins into breast milk is scarce. Some case studies have observed the presence of rosuvastatin and atorvastatin in breast milk; however, the concentrations were low, and the infant exposure was thus assumed to be low.^12^,^14^ More studies on the concentration of statins in breast milk are therefore needed.

In this study, we aim to increase the knowledge on the lipid profile during breastfeeding in women with and without FH, and the concentration of statins in breast milk.

## Methods and analysis

### Study aims

The overall aim of the FH-FEMINA study is to investigate the change in lipid profile in the first year postpartum in women with and without FH. We will also investigate the association between breastfeeding and other cardiovascular risk markers, as well as the concentration of statins in breast milk.

### Primary outcome

In women with FH compared with women without hypercholesterolaemia, we will investigate

1. The change in serum lipid profile (total cholesterol, LDL-C, high-density lipoprotein cholesterol (HDL-C), triglycerides, lipoprotein(a)) from 2 to 4 weeks post partum up to 12 months postpartum (absolute and relative change).

### Secondary outcomes

In women with FH compared with women without hypercholesterolaemia, we will investigate

1. The association between breastfeeding (yes/no, exclusive/partial, frequency and duration) and additional markers for ASCVD risk, eg,
  - Inflammatory markers (eg, C-reactive protein (CRP)
  - Metabolic markers (eg, glucose)
  - Weight
  Measurements will be performed in both plasma/serum and in peripheral blood mononuclear cells (PBMCs) (gene expression levels).
2. The breast milk composition (lipids and metabolites) throughout the breastfeeding period.

In only women with FH, we will investigate

1. The concentration of statins in the breast milk and dried blood spot of the offspring of women with FH after start of lipid-lowering medication.
2. The concentration of total cholesterol and metabolic markers in dried blood spots collected at 12 months postpartum in the offspring of women with FH.

### Study sample

#### Women with FH

We aim to recruit a total of 50 pregnant women with FH across three countries (Norway, the Netherlands and the Czech Republic). Inclusion criteria for women with FH are (1) age 18 years or older, (2) confirmed diagnosis of FH through genetic testing or clinical assessment based on the Dutch Lipid Clinic Network^2^, (3) singleton pregnancy in the third trimester and (4) a sufficient command of Norwegian, Dutch, Czech or English language. Pregnant women with FH or women with FH planning to become pregnant will be informed about the study via outpatient lipid clinics in Norway, the Netherlands and the Czech Republic. In addition, patients will be recruited through advertisements via applicable national patient organisations, colleague healthcare professionals in the three countries and via social media platforms.

#### Women without hypercholesterolaemia (control group)

In Norway, 20 pregnant women will be included in a control group. Inclusion criteria for women without hypercholesterolaemia are (1) age 18 years or older, (2) singleton pregnancy in the third trimester and (3) a sufficient command of the Norwegian or English language. Exclusion criteria for the control group are (1) known pre-pregnancy hypercholesterolaemia, (2) a history of ASCVD, (3) pre-eclampsia or gestational diabetes and (4) pre-pregnancy body mass index (BMI) <18 or >30 kg/m^2^. The women in the control group will be recruited via advertisements on social media and via the University of Oslo and Oslo University Hospital.

### Study design and setting

The study will be a prospective study carried out in Norway, the Netherlands and the Czech Republic. The women will be included at 36 weeks gestation until 12 months post partum or to the end of the breastfeeding period if the participant is breastfeeding for more than 12 months. The study inclusion started in June 2022, with recruitment planned to conclude by the second quarter half of 2025. The data collection phase will end when the last participant has completed all applicable study visits, estimated to be by the end of 2026.

#### Study visits

**Figure 1:**
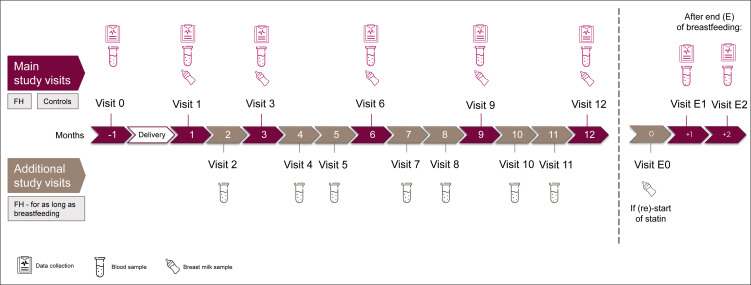
Study design. FH, familial hypercholesterolaemia.

Women are included in gestational week 36 (V0) and followed up at the main study visits (figure 1) scheduled at 2–4 weeks postpartum (V1), and 3 (V3), 6 (V6), 9 (V9) and 12 (V12) months postpartum and 1 (VE1) and 2 months (VE2) after the end of breastfeeding. At all these main study visits, a non-fasting venous blood sample, breast milk samples (for as long as breastfeeding) and information on health, diet and medical factors are collected (table 1). At additional study visits among the women with FH, for as long as they are breastfeeding, blood samples will be collected at 2 (V2), 4 (V4), 5 (V5), 7 (V7), 8 (V8), 10 (V10) and 11 (V11) months postpartum. These blood samples can be collected at a time of the women’s choice within the time window for the study visit at the blood drawing location at the hospital, laboratory or GP office. At the additional visits, information on breastfeeding status and maternal weight will also be collected.

**Table 1:** Data collection at study visits

Study visit (months)	Medical history, lifestyle factors	Breastfeeding status Maternal weight	Blood pressure	Dietary data	Offspring weight and length	Blood sample, routine measurements	Blood sample, additional measurements	Breast milk sample
**V0 (−1)**	X	X	X	X		X	X	
**V1 (1)**		X	X	X	X	X	X	X
V2 (2)		X				X		
**V3 (3)**		X	X	X	X	X	X	X
V4 (4)		X				X		
V5 (5)		X				X		
**V6 (6)**		X	X	X	X	X	X	X
V7 (7)		X				X		
V8 (8)		X				X		
**V9 (9)**		X	X	X	X	X	X	X
V10 (10)		X				X		
V11 (11)		X				X		
**V12 (12)**		X	X	X	X	X	X	X
VE0								
**VE1 (+1)**		X	X	X		X	X	X
**VE2 (+2)**		X	X	X		X	X	X

**Main study visits** (FH and controls): V0, V1, V3, V6, V9, V12, VE1, VE2.

Additional study visits (FH, for as long as breastfeeding): V2, V4, V5, V7, V8, V10, V11, VE0.

FH, familial hypercholesterolaemia.

In Norway, study visits will take place at the University of Oslo and at the outpatient lipid clinics in Trondheim, Bergen and Bodø. To increase participation from women living outside the larger cities in Norway, women will also be followed at digital study visits with blood samples collected at their local GP office. In the Netherlands, all study visits and blood sampling will be performed at the Erasmus University Medical Center in Rotterdam. In the Czech Republic, study visits will take place at four study centres in Prague, Brno, Hradec Kralove and Zlin.

### Outcomes

#### Medical history, lifestyle factors and clinical examinations

At the first study visit, information on medical history, including FH diagnosis (age, genetic/clinical), lipid-lowering treatment history, untreated lipid levels prior to pregnancy, use of other medication and dietary supplements, history of current and previous pregnancies, lifestyle factors (physical activity, diet and smoking) and education level, will be collected. At all main study visits (table 1), information on delivery and any complications during pregnancy or delivery, breastfeeding status (yes/no, exclusive/partial, and frequency), BMI and blood pressure are collected. Dietary data are collected at the end of pregnancy and 12 months post partum using a digital food frequency questionnaire to capture the habitual diet during pregnancy and breastfeeding. Dietary data are also collected at all main study visits using a 24-hour dietary recall interview or questionnaire to capture detailed information on the diet the days prior to the blood sample. Weight and length of the child will be collected at the main study visits. At the additional visits, breastfeeding status and frequency, and self-reported maternal weight will also be collected (table 1).

#### Blood samples

A non-fasting venous blood sample will be collected at the main visits and additional visits for as long as the women are breastfeeding (table 1). The following measurements will be performed: lipid levels (total cholesterol, LDL-C, HDL-C, triglycerides, apolipoprotein A1, apolipoprotein B, lipoprotein(a)), glucose, HbA1c, c-peptide, creatinine, albumin, alanine transaminase, aspartate transaminase, thyroid-stimulating hormone, free T4, white blood cell count, high-sensitivity CRP (hs-CRP), haemoglobin, ferritin, vitamin D, folate, vitamin B_12_ and fatty acid composition. At the main study visits, the following measurements will also be performed: (1) targeted serum metabolomic profiling, including lipoprotein profiling, amino acids and glucose metabolites; (2) circulating inflammatory markers (such as CRP, interleukin (IL)-6, IL-10, tumour necrosis factor-α and interferon-γ); (3) in the Oslo and Rotterdam study centres only: PBMC gene expression of inflammatory and lipid-related genes in order to identify the differences in metabolic pathways influenced by breastfeeding. Non-fasting blood samples are used to minimise the burden for the participants in this study as these measures show minimal changes in response to normal food intake.^15 16^

#### Breast milk samples

Breast milk samples will be collected at the main study visits (table 1) for as long as the women are breastfeeding. Main outcome measures in the breast milk samples are lipid profiling (lipidomics) and metabolite profiling (metabolomics).

If the women with FH (re)-start lipid-lowering treatment (eg, statin) while still having breast milk production, additional breast milk samples will be collected. This breast milk sampling will be standardised by collecting the breast milk samples on days 0, 1, 2 and 3 after statin initiation. For day 0, 1 and 2, a single breast milk sample is collected between 06:00-12:00. On day 3, when the statin is expected to have reached a steady state in breast milk, multiple breast milk samples will be collected at 0, 2, 5 and 12 hours after intake of the statin pill. For all breast milk samples, the participants are asked to pump one breast from full to empty. The concentration of statins will be measured in these breast milk samples. We expect to collect fewer breast milk samples from this time point as it may be challenging to synchronise the initiation of lipid-lowering treatment with the end of breastfeeding.

#### Dried blood spot samples

A dried blood spot sample (DBS) from the offspring of women with FH will be collected at 12 months postpartum if informed consent has been given. Lipid profile and other metabolic markers (eg, inflammatory markers) will be analysed in the DBS of the offspring to assess associations with maternal levels. Additionally, we will investigate the association between offspring lipid levels and other metabolic markers with the duration of breastfeeding, as well as the composition of breast milk.

If breast milk samples are collected at the start of lipid-lowering treatment, the women with FH will also be invited to take a DBS of themselves and their child (provided that informed consent has been given) to measure the concentration of the lipid-lowering drug in the DBS samples.

### Safety plan

A 30–50% increase in cholesterol levels during pregnancy is normal and expected, but there is limited knowledge available about when and how fast the cholesterol levels decrease after delivery. Women are not routinely monitored during the breastfeeding period. Therefore, the GP or treating physician at a lipid clinic of the women with FH will be informed about the participant’s lipid levels (total cholesterol, LDL-C, HDL-C and triglycerides) from the blood sample taken at 9 months postpartum (V9) to ensure that the GP or treating physician will evaluate further treatment for the woman.

### Sample size calculation

Sample size calculation for comparing the difference between the two groups (women with FH and control women without hypercholesterolaemia) in changes from 2 to 4 weeks to 6 months postpartum was performed for LDL-C (mmol/L) with 5% significance level and 80% power. Based on the experiences from previous studies, availability of pregnant women and motivation to participate, the project group does not find it realistic to include more than 50 women with FH and 20 control women, that is, a ratio of 2.5 between the sample sizes.

Amundsen *et al*^6^ studied changes during pregnancy in women with FH (n=22) and women without hypercholesterolaemia (n=149). Mean LDL-C in gestational week 36 in the two groups was 8.6 and 3.9, mmol/L, respectively. In the women with FH, mean LDL-C at 3–6 months post partum was 7.0 (n=17), that is, mean change of 1.6 mmol/L, but no information was given about postpartum LDL-C values in the women without hypercholesterolaemia. Fahraeus *et al*^17^ observed LDL-C at 2 months postpartum in healthy women (n=14) and the mean was 3.51 mmol/L. However, we are not aware of the results in the literature reporting SDs for the changes in LDL-C during breastfeeding for both women with and without FH.

A difference in LDL-C of 0.43 mmol/L (16.7 mg/dL) was shown to be associated with reduced occurrence of coronary heart disease in the IMPROVE-IT study.^18^ Thus, we consider a difference in LDL-C of 0.8 mmol/L to be a clinically relevant difference between the two groups (women with FH and control women) in changes from 2 to 4 weeks to 6 months postpartum. With a 5% level of significance, 80% power and a 2.5 ratio in sample size, we need 45 women with FH and 18 control women to observe a difference in changes of 0.8 mmol/L assuming an SD of 1.0 mmol/L in each group. Thus, we will include 50 women with FH and 20 control women.

### Statistical analysis

Descriptive data will be expressed as mean (SD), median (IQR or min-max) or frequency (percentage). Mixed models for repeated measurements will be used to study the changes over time within and between the two groups. Missing data will be described, and mixed models for repeated measurements handle missing data.^19^ Potential confounding variables will be evaluated by directed acyclic graphs and included in the model. Depending on the amount of missing data in the confounding variables, multiple imputation will be considered.

### Patient and public involvement

Patient organisation was involved in the recruitment process. Patients and/or the public were not involved in the design, or conduct, or reporting, or dissemination plans of this research.

## Ethics and dissemination

In all participating centres, ethical approval shall be obtained prior to study start (Norway: Regional Committees for Medical Research Ethics South-East Norway, No. 395816; the Netherlands: MEC-2024-0139; the Czech Republic: 145/23 S-Grant). The FH-FEMINA study is registered on ClinicalTrials.gov, #NCT05367310. All possible participants will receive ample time to read the informed consent forms prior to the first study visit and will have time to ask questions before signing the informed consent form.

The findings from this research will be disseminated to stakeholders, such as healthcare professionals, researchers and patients, via peer-reviewed scientific article(s), presentations at conferences, patient organisation meetings and social media.

Frequent blood sampling and breast milk collection may pose a burden to participants. There is a significant knowledge gap about the lipid profile the first year postpartum in women with FH compared with women without FH. To minimise participant burden, we have carefully optimised blood collection volumes in collaboration with the laboratories to maximise the information obtained while minimising the volume required. Breast milk samples are collected every 3 months postpartum during breastfeeding. Recognising that pumping of breast milk may depend on many factors, we emphasise to participants that participation is voluntary and that there are no consequences if breast milk samples cannot be provided. Participants are explicitly informed that the child’s needs take precedence, and collection is only encouraged in the case of surplus breast milk production. This study aims to provide valuable insights into lipid changes in relation to breastfeeding in women with FH during the postpartum period, with the goal of improving care and guidance for women with FH in the future.

## Discussion

For women with FH, the life stages of pregnancy and breastfeeding result in periods of interrupted treatment. Currently, there are no established strategies in existing guidelines on how to address or compensate for these off-treatment periods in the years before and after childbearing in women with FH. Whether these periods of increased cholesterol exposure contribute to a higher cardiovascular risk in women with FH remains to be investigated.

In this study, we will closely monitor the lipid profile during breastfeeding, with frequent blood samples for as long as the women are breastfeeding. We will thereby obtain detailed data on the changes in the lipid profile during the breastfeeding period in women with FH compared with women without hypercholesterolaemia.

The evidence in the literature on the transfer of statins in breast milk is scarce. Therefore, more data on the concentration of statins in breast milk are needed to better advise women with FH and provide the best care for this patient group during the childbearing years.

There are several benefits of breastfeeding for both the mother and child, including both metabolic and psychological aspects. The aim of this study is to increase the knowledge on breastfeeding in FH to further improve the information and treatment for women at high risk of ASCVD. This could in turn contribute to the establishment of specific guidelines for women with FH. Future guidelines should include strategies on how to compensate for the lost treatment time during pregnancy and breastfeeding before and after childbearing age in women with FH.
