# Supplementary material for: Prospective study on breastfeeding, lipid profile and cardiovascular risk markers in women with familial hypercholesterolaemia: study protocol for the FH-FEMINA study
Source: BMJ Open. 2025 Apr 27;15(4):e092208. doi: 10.1136/bmjopen-2024-092208 (PMC12035426; doi:10.1136/bmjopen-2024-092208)
Supplement: Supplementary file 1 [file bmjopen-2024-092208.reviewer_comments.pdf]

PEER REVIEW HISTORY

BMJ Open publishes all reviews undertaken for accepted manuscripts. Reviewers are asked to complete a checklist review form and are provided with free text boxes to elaborate on their assessment. These free text comments are reproduced below.

ARTICLE DETAILS

Title (Provisional)

Study protocol for the FH-FEMINA study: a prospective study on breastfeeding, lipid profile and cardiovascular risk markers in women with familial hypercholesterolemia

Authors

Klevmoen, Marianne; Mulder, Janneke; Prøven Bogsrud, Martin; Retterstøl, Kjetil; Vesterbekkmo, Elisabeth K; Pedersen, Eva K.R.; Freiberger, Tomas; Vrablik, Michal; Vaclova, Martina; Hovland, Anders; Vethe, Nils T; Brekke, Hilde Kristin; Iversen, P O; Veierød, Marit; Roeters van Lennep, Jeanine; Holven, KIRSTEN B. B

VERSION 1 - REVIEW

**Reviewer**	**1**
**Name**	**Graham, Dorothy**
**Affiliation**	
**Date**	**02-Sep-2024**
**COI**	**none**

I congratulate you on planning this important study. Hopefully it will provide evidence to assist women with FH make more informed decisions regarding breastfeeding plans.

**Reviewer**	**2**
**Name**	**Osman, Doaa A.**
**Affiliation**	**Department of Physical Therapy for Woman's Health, Cairo University, Physical Therapy for Woman's Health**
**Date**	**21-Nov-2024**
**COI**	**I declare no competing interests related to the review of this manuscript.**

Study protocol for the FH-FEMINA study: a prospective study on breastfeeding, lipid profile and cardiovascular risk markers in women with familial hypercholesterolemia

Thank you for the opportunity to review this manuscript. The study protocol addresses a significant gap in understanding the effects of breastfeeding on lipid profiles and cardiovascular risk markers in women with familial hypercholesterolemia (FH). It also investigates the transfer of statins into breast milk, which is a novel and clinically relevant topic. However, several areas require clarification and improvement:

1. Abstract:

Strengths and limitations of this study:

• While the authors acknowledge the small sample size as a limitation, they should consider discussing potential strategies to mitigate this, such as extending the recruitment period or expanding to additional study sites.

2. Methods and analysis:

Sample Size Calculation:

• Please, add the exclusion criteria for women with FH.

• The control group is only recruited in Norway. Consider including control participants from all three countries to improve comparability.

• Provide a more detailed explanation or reference for why a difference of 0.8 mmol/L is clinically meaningful.

Outcomes: Breast milk samples

• If the women with FH (re)-start lipid-lowering treatment (e.g., statin) while still having breast milk production, additional breast milk samples will be collected according to a standardized protocol. How will the breast milk sampling be standardized?

Outcomes: Dried blood spot samples

• The focus on offspring outcomes (e.g., dried blood spot analysis) is intriguing but underdeveloped. Please, provide more detail on why this outcome is relevant to the study aims.

Statistical analysis:

• The statistical analysis plan could be more detailed, including specific methods for handling missing data and potential confounding factors.

3. Ethical considerations:

• Elaborate on any potential risks or burdens to participants, especially regarding the frequent blood sampling and breast milk collection.

4. Discussion:

• Add the discussion of study limitations and potential confounders, such as variability in breastfeeding practices or compliance with statin re-initiation protocols.

**Reviewer**	**3**
**Name**	**Wallace, McKenzie**
**Affiliation**	**The Ohio State University**
**Date**	**26-Nov-2024**
**COI**	**I have no conflicts of interest**

Thank you for the opportunity to review this work. This study will provide critical information about cholesterol level changes in women with FH. I have a few comments and questions to improve reporting of the study methods.

Introduction:

The introduction covers several topics making it a bit difficult to understand the purpose. I recommend content be limited to the direct concepts of the study (FH, cholesterol, breastfeeding, statin use)

Methods:

The verbatim study aims are confusing when all of the details on what will be measured aren't included. A brief summary of the aims as a purpose statement would be more understandable

Throughout the methods section many sections can be combined into fewer paragraphs

What is the rational for the control group only coming from Norway? Also will experiencing a pregnancy complication like preeclampsia or gestational diabetes in the control pregnancy also be an exclusion criteria?

If a primary goal of the study is to investigate cholesterol levels, why are non-fasting samples being obtained? This seems to be a major concern

It's difficult to read the outcome sections with the V0, V1, etc in the sentences. A table would be a better way to display what is collected when and then remove V0, V1 etc from the outcomes section. Or this information can be added to the figure.

Under dietary data, are the data being collected two different ways? Authors mention both FFQ and 24 hour recall. One method would make for more comparable data. Please clarify rational

What inflammatory markers are being assessed? CRP is mentioned in multiple places, but are other markers being measured?

Why are some markers only assessed at some locations?

Breastmilk in treatment group- what is meant by a standardized protocol? How is it different than breastmilk samples obtained during other study visits?

Is there any feasibility data for obtaining lipids and metabolic markers from dried blood spots?

Sample size- what is the rational for 0.8mmol/L to be clinically significant?

Safety plan- Samples taken at 9 months will be reported to GP. So are these samples being processed immediately after being obtained? Rather than a batch analysis?

**Reviewer**	**4**
**Name**	**Shub, Alexis**
**Affiliation**	**Mercy Hospital for Women**
**Date**	**30-Nov-2024**
**COI**	**None**

Thank you for the opportunity to review this important project which will provide meaningful information about breastfeeding on medication, and about disease status in women with FH, and so enable women to be make informed choices

My only question is that given the uncommon nature of the disease, and likely difficulty recruiting, could the authors please explain why they have decided that multiple pregnancy is an exclusion. The information about medication in breast milk will still be valid in these women, and as all the other investigations are post partum, they should also be valid

---

VERSION 1 - AUTHOR RESPONSE

Reviewer: 2

Thank you for the opportunity to review this manuscript. The study protocol addresses a significant gap in understanding the effects of breastfeeding on lipid profiles and cardiovascular risk markers in women with familial hypercholesterolemia (FH). It also investigates the transfer of statins into breast milk, which is a novel and clinically relevant topic. However, several areas require clarification and improvement:

1. Abstract:
   Strengths and limitations of this study:
   While the authors acknowledge the small sample size as a limitation, they should consider discussing potential strategies to mitigate this, such as extending the recruitment period or expanding to additional study sites.

   We thank the reviewer for highlighting this important aspect. Unfortunately, due to budgetary constraints, it is not feasible to expand beyond the existing study sites in Norway, the Netherlands, and the Czech Republic. However, we have decided to extend the recruitment

period until the second half of 2025.
We acknowledge that the sample size remains a limitation given the small population of pregnant women with FH available during the recruitment period. We are actively working to achieve the minimum number of women with FH required, as determined by our sample size calculation.

2. Methods and analysis:
   Sample Size Calculation:
   - Please, add the exclusion criteria for women with FH.
     Thank you for this comment. The exclusion criteria for women with FH are implicitly covered by the inclusion criteria. The inclusion criteria for women with FH are: 1) age 18 years or older, 2) confirmed diagnosis of FH through genetic testing or clinical assessment based on the Dutch Lipid Clinic Network (3)), 3) singleton pregnancy in the third trimester, and 4) a sufficient command of Norwegian, Dutch, Czech or English language. For this study, the exclusion criteria are therefore the reverse of the inclusion criteria: 1) age below 18 years, 2) not diagnosed with FH, 3) multiple pregnancy, 4) insufficient command of the Norwegian, Dutch, Czech or English language. Since women who do not meet the inclusion criteria are automatically excluded from the study, we have not explicitly stated these exclusion criteria in the manuscript.

   - The control group is only recruited in Norway. Consider including control participants from all three countries to improve comparability.
     We appreciate this comment. We agree that including control participants from all three countries would improve the study. However, logistical and practical constraints (e.g. budgetary, personnel, etc.) have limited our ability to recruit control participants in the Netherlands and Czech Republic. Despite this limitation, we believe that the general population (control group) across the three European countries shares similar baseline characteristics, as previously seen in a study from Norway and the Netherlands (1). Consequently, we do not anticipate significant differences in lipid profiles, which is the primary objective of our study. Additionally, the majority of the included women with FH are expected to be Norwegian (estimated to comprise at least 60% of the study sample). We acknowledge this as a limitation of our study and have described this further in the "Strengths and limitations" section after the abstract, page 4.

   - Provide a more detailed explanation or reference for why a difference of 0.8 mmol/L is clinically meaningful.
     We thank the reviewer for the opportunity to clarify this. Very little data exists on the lipid profile during pregnancy among healthy women compared to women with FH. A difference in LDL-C of 0.43 mmol/L (16.7 mg/dL) was shown to be associated with reduced occurrence of coronary heart disease in the IMPROVE-IT study (2). Thus, a difference of 0.8 mmol/L during a breastfeeding period from 2-4 weeks to 6 months postpartum between women with and without FH would be considered clinically relevant. We have now included this information in the revised manuscript, page 11 line 19-20.

3. Outcomes: Breast milk samples

If the women with FH (re)-start lipid-lowering treatment (e.g., statin) while still having breast milk production, additional breast milk samples will be collected according to a standardized protocol. How will the breast milk sampling be standardized?

We thank the reviewer for this comment and appreciate the opportunity to provide further clarification on the standardization of breast milk collection at the time of (re-)starting lipid-lowering treatment. Breast milk sampling is standardized as follows: breast milk samples are collected on Day 0, Day 1, Day 2, and Day 3 after statin initiation. For Day 0, Day 1, and Day 2, a single breast milk sample is collected between 06:00-12:00. On Day 3, when the statin is expected to have reached a steady state in breast milk, multiple breast milk samples will be collected at 0, 2, 5, and 12 hours after intake of the statin pill. For all breast milk samples, the participants are asked to pump one breast from full to empty.
We have now included these details in the protocol regarding the breast milk sampling at statin (re)initiation in the methods section, page 10 lines 8-13 of the revised manuscript.

4. Outcomes: Dried blood spot samples
   The focus on offspring outcomes (e.g., dried blood spot analysis) is intriguing but underdeveloped. Please, provide more detail on why this outcome is relevant to the study aims.

   We appreciate this comment from the reviewer.
   Currently, little is known about the impact of having a mother with FH on offspring during this early stage of life. This includes uncertainties surrounding cholesterol levels and cardiovascular risk, as addressed in a recent review (3). The FH-FEMINA study aims to address these gaps by gaining insights into the lipid profiles of offspring in relation to maternal cholesterol levels in women with FH during the first postpartum year, as well as breastfeeding status and breast milk composition.
   Understanding the benefits and potential disadvantages of lipid-lowering treatment for both mother and child is crucial when managing women with FH during pregnancy and the first postpartum year. For this reason, we have also included child-related outcomes in our study. Specifically, we will use the dried blood spot samples to measure total cholesterol and other lipid and metabolic markers such as inflammatory markers to assess the association between maternal and offspring levels. Additionally, we will assess the association between offspring cholesterol level and the duration of breastfeeding, as well as the composition of breast milk.
   We have now included additional information about the offspring dried blood spot measurements in the revised manuscript, page 10 lines 20-22.

5. Statistical analysis:
   The statistical analysis plan could be more detailed, including specific methods for handling missing data and potential confounding factors.
   We thank the reviewer for this comment. Missing data will be described, and the mixed models for repeated measurements handle missing data (4). Potential confounding variables will be evaluated by directed acyclic graphs and included in the model. Depending on the amount of missing data in the confounding variables, multiple imputation will be considered.
   We have now included information on handling of missing data and potential confounding factors in the revised manuscript, page 11-12 lines 30-2.

6. Ethical considerations:
   Elaborate on any potential risks or burdens to participants, especially regarding the frequent blood sampling and breast milk collection.

   We appreciate the reviewer's comment and acknowledge that frequent blood sampling and breast milk collection may pose a burden to participants.
   There is a significant knowledge gap regarding the care of women with FH during and around pregnancy. To date, the study by Amundsen et al. (5) is the only study available in the literature on cholesterol profiles in women with FH during the first year postpartum. The study of Amundsen et al. primarily focused on cholesterol changes during pregnancy and provided only a single measurement between 3-6 months postpartum, leaving the trajectory of lipid normalization in women with FH unclear (5). In the general population, lipid levels generally stabilize to baseline levels by 4 months postpartum (6), but this timeline may differ in women with FH. Given the high inter-individual variability in LDL-C levels among women with FH, influenced by their specific pathogenic variants, repeated measurements in the same woman are essential to understand the trajectory of LDL-C levels postpartum.
   To minimize participant burden, we have carefully optimized blood collection volumes in collaboration with the laboratories to maximize the information obtained while minimizing the volume required, ensuring that we only collect what is essential. If breastfeeding ends before 12 months postpartum, only the main study visits (3, 6, 9, and 12 months) are conducted after end of breastfeeding. In the additional visits, only routine measurements are collected, and thus fewer tubes are collected.
   Breast milk samples are collected every three months postpartum during breastfeeding. Recognizing that pumping of breast milk may depend on many factors, we emphasize to participants that participation is voluntary and has no consequences if breast milk samples cannot be provided. Participants are explicitly informed that the child's needs take precedence, and collection is only encouraged in the case of surplus breast milk production. This study aims to provide valuable insights into lipid changes in relation to breastfeeding in women with FH during the postpartum period, with the goal of improving care and guidance for women with FH in the future. We have now elaborated more on this in the revised manuscript, page 12, line 14-25.

7. Discussion:
   Add the discussion of study limitations and potential confounders, such as variability in breastfeeding practices or compliance with statin re-initiation protocols.

   We thank the reviewer for this comment. We agree that variability in breastfeeding practices and other potential confounders are critical aspects to consider as study limitations. We acknowledge that the timing of statin re-initiation following breastfeeding cessation may vary among participants. We have previously observed differences in duration of breastfeeding in Norway (median 8.5 months) and the Netherlands (median 3.6 months) (1). We have included variability in breastfeeding practices in the Strengths and limitations section page 4. Regarding compliance with statin re-initiation protocols, we acknowledge the lack of standardized guideline recommendations regarding breastfeeding and statin reinitiation, which stems from insufficient data on the topic. Consequently, we have not framed this as a limitation per se, but we emphasize the need for further research to develop evidence-based recommendations for care of women with FH during and after pregnancy.

Reviewer: 3

Thank you for the opportunity to review this work. This study will provide critical information about cholesterol level changes in women with FH. I have a few comments and questions to improve reporting of the study methods.

Introduction:

The introduction covers several topics making it a bit difficult to understand the purpose. I recommend content be limited to the direct concepts of the study (FH, cholesterol, breastfeeding, statin use)

We thank the reviewer for this comment to improve the readability of the introduction. We have shortened the introduction to focus more specifically on the key topics relevant to the study (page 5-6).

Methods:

- The verbatim study aims are confusing when all of the details on what will be measured aren't included. A brief summary of the aims as a purpose statement would be more understandable
  We appreciate the feedback and have now included a summary of the aims in the manuscript, page 7 line 3-6.

- Throughout the methods section many sections can be combined into fewer paragraphs
  We appreciate this comment from the reviewer. We have revised the methods section and combined it into fewer paragraphs (pages 7-10).

- What is the rational for the control group only coming from Norway? Also will experiencing a pregnancy complication like preeclampsia or gestational diabetes in the control pregnancy also be an exclusion criteria?
- **We appreciate this comment. We agree that including control participants from all three countries would improve the study. However, logistical, and practical constraints (e.g. budgetary, personnel, etc.) have limited our ability to recruit control participants in the Netherlands and Czech Republic. Despite this limitation, we believe that the general population (control group) across the three European countries shares similar baseline characteristics (1). Consequently, we do not anticipate significant differences in lipid profiles, which is the primary objective of our study. Additionally, the majority of the included women with FH are expected to be Norwegian (estimated to comprise at least 60% of the study sample). We acknowledge this as a limitation of our study and have described this further in the "Strengths and limitations" section after the abstract, page 4. We also agree that preeclampsia or gestational diabetes can influence the study outcomes in the control group. We have now included this, as well as BMI <18 or >30 kg/m^2, as exclusion criteria for the control group, as all these factors may influence the lipid metabolism in the control group, on page 8 line 6-7.**

- If a primary goal of the study is to investigate cholesterol levels, why are non-fasting samples being obtained? This seems to be a major concern
  We thank the reviewer for the opportunity to clarify the rationale for using non-fasting samples. As the primary objective of this study is to investigate cholesterol levels (total cholesterol, LDL-cholesterol, HDL-cholesterol), we chose non-fasting blood samples based on research showing that these measures show minimal changes in response to normal food intake (7, 8). Given the frequent blood sampling required in this study, we use non-

fasting blood samples to minimize the burden for the participants. Also, as the study sample consists of breastfeeding women, obtaining repeated fasting blood samples would impose an additional burden on the participants. We have now included this in the revised manuscript page 9-10 line 31-2.

- It's difficult to read the outcome sections with the V0, V1, etc in the sentences. A table would be a better way to display what is collected when and then remove V0, V1 etc from the outcomes section. Or this information can be added to the figure.
We appreciate this comment from the reviewer and acknowledge that the outcomes section may be difficult to read. We have now removed V0, V1 etc. from the sentences in the outcome sections (pages 9-10) and added information about the data collected at each visit in a new Table 1 in the revised manuscript.

- Under dietary data, are the data being collected two different ways? Authors mention both FFQ and 24 hour recall. One method would make for more comparable data. Please clarify rational
We use two dietary data collection methods, the Food Frequency Questionnaire (FFQ) and 24-hour recall, as they capture complementary aspects of dietary intake. The FFQ, collected at gestational week 36 and 12 months postpartum, provides information on the habitual diet over the past year. In contrast, the 24-hour recall, collected at all main study visits, captures detailed information on the dietary intake in the days prior to the study visit and therefore could describe whether the diet may influence the lipid levels measured at the main study visit. This dual approach allows us to assess both long-term dietary habits and short-term dietary factors that could influence lipid levels measured at the study visits. We have included more information about the dietary data being collected in the revised manuscript (page 9, lines 13-16).

- What inflammatory markers are being assessed? CRP is mentioned in multiple places, but are other markers being measured?
In addition to CRP, we will measure inflammatory markers, such as IL-6, IL-10, TNF-α, and IFN-γ. These measurements will depend on the analysis platform chosen, as they often provide specific inflammation panels. We have now added this information in the manuscript (page 9, line 29).

- Why are some markers only assessed at some locations?
We thank the reviewer for this comment. The samples for PBMC gene expression are collected only at the Oslo and Rotterdam study centres due to logistical constraints, which made it unfeasible to include this analysis at all study centres.

- Breastmilk in treatment group- what is meant by a standardized protocol? How is it different than breastmilk samples obtained during other study visits?
We thank the reviewer for this comment and appreciate the opportunity to provide further clarification on the standardization of breast milk collection at the time of (re-)starting lipid-lowering treatment. Breast milk sampling is standardized as follows: breast milk samples are collected on Day 0, Day 1, Day 2, and Day 3 after statin initiation. For Day 0, Day 1, and Day 2, a single breast milk sample is collected between 06:00-12:00. On Day 3, when the statin is expected to have reached a steady state in breast milk, multiple breast milk samples will be collected at 0, 2, 5, and 12 hours after intake of the statin pill. For all breast milk samples, the participants are asked to pump one breast from full to empty.

We have now included these details in the protocol regarding the breast milk sampling at statin (re)initiation in the methods section, page 10 lines 8-13, of the revised manuscript.

- Is there any feasibility data for obtaining lipids and metabolic markers from dried blood spots?
  Our research groups have previously demonstrated a strong correlation between total cholesterol levels measured in dried blood spots and venous blood samples (9, 10).

- Sample size- what is the rational for 0.8mmol/L to be clinically significant?
  We thank the reviewer for the opportunity to clarify this. Very little data exists on the lipid profile during pregnancy among healthy women compared to women with FH. A difference in LDL-C of 0.43 mmol/L (16.7 mg/dL) was shown to be associated with reduced occurrence of coronary heart disease in the IMPROVE-IT study (2). Thus, a difference of 0.8 mmol/L during a breastfeeding period from 2-4 weeks to 6 months postpartum between women with and without FH would be considered clinically relevant. We have now included this information in the revised manuscript, page 11 line 19-20.

- Safety plan- Samples taken at 9 months will be reported to GP. So are these samples being processed immediately after being obtained? Rather than a batch analysis?
  The routine measurements collected at all study visits such as total cholesterol, LDL-C, HDL-C, and triglycerides will be analysed continuously after being obtained and lipid profile from 9 months will thus be reported to GP or treating physician after the visit. The other analyses collected at the main study visits (targeted serum metabolomic profiling, circulating inflammatory markers, and PBMC gene expression) will be analysed in a batch at end of study.
  We acknowledge that having the routine measurements analysed continuously after being obtained may result in measurement variability and we acknowledge this as a limitation of the study.

Reviewer: 4

Thank you for the opportunity to review this important project which will provide meaningful information about breastfeeding on medication, and about disease status in women with FH, and so enable women to be make informed choices

My only question is that given the uncommon nature of the disease, and likely difficulty recruiting, could the authors please explain why they have decided that multiple pregnancy is an exclusion. The information about medication in breast milk will still be valid in these women, and as all the other investigations are post partum, they should also be valid

We thank the reviewer for this comment. The inclusion of women with multiple pregnancies would introduce potential biases and would complicate data analysis and interpretation for several reasons. The composition of breast milk is known to differ in women with multiple pregnancies compared to singletons (11). Although there is no direct evidence, to our knowledge, that medication concentrations differ in breast milk between these groups, these compositional differences could introduce confounding factors.
Also, we cannot rule out that lipid levels during pregnancy could be different in women with multiple gestation compared to women with a singleton pregnancy. Although limited research exists on this topic, postpartum recovery may also differ (e.g. prolonged metabolic recovery and greater weight retention potentially influencing lipid normalization), and variability in

breastfeeding practices could further confound lipid profiles postpartum, complicating the interpretation of study outcomes.
Furthermore, a small percentage of all live births are multiple pregnancies (1.4% in 2023 in Norway) (12). The likelihood of recruiting women with FH who also have a multiple pregnancy is therefore extremely low. Although they represent an interesting group, it would make it impractical to recruit a sufficient sample size for a meaningful analysis.
Finally, up to 60% of twin pregnancies deliver before gestational week 37 (13), which could affect the possibility for participants to attend the study visit in gestational week 36.
Therefore, to minimize the risk of missed study visits and maximize comparability, we only include women with singleton pregnancies for a more homogenous and comparable study sample.

References

1. Klevmoen M, Bogsrud MP, Retterstøl K, Svilaas T, Vesterbekkmo EK, Hovland A, et al. Loss of statin treatment years during pregnancy and breastfeeding periods in women with familial hypercholesterolemia. Atherosclerosis. 2021;335:8-15.
2. Cannon CP, Blazing MA, Giugliano RP, McCagg A, White JA, Theroux P, et al. Ezetimibe Added to Statin Therapy after Acute Coronary Syndromes. The New England journal of medicine. 2015;372(25):2387-97.
3. Mulder J, Kusters DM, Roeters van Lennep JE, Hutten BA. Lipid metabolism during pregnancy: consequences for mother and child. Curr Opin Lipidol. 2024;35(3):133-40.
4. Twisk J, de Boer M, de Vente W, Heymans M. Multiple imputation of missing values was not necessary before performing a longitudinal mixed-model analysis. J Clin Epidemiol. 2013;66(9):1022-8.
5. Amundsen AL, Khoury J, Iversen PO, Bergei C, Ose L, Tonstad S, et al. Marked changes in plasma lipids and lipoproteins during pregnancy in women with familial hypercholesterolemia. Atherosclerosis. 2006;189(2):451-7.
6. Wiznitzer A, Mayer A, Novack V, Sheiner E, Gilutz H, Malhotra A, et al. Association of lipid levels during gestation with preeclampsia and gestational diabetes mellitus: a population-based study. Am J Obstet Gynecol. 2009;201(5):482.e1-8.
7. Langsted A, Nordestgaard BG. Nonfasting versus fasting lipid profile for cardiovascular risk prediction. Pathology. 2019;51(2):131-41.
8. Nordestgaard BG, Langsted A, Mora S, Kolovou G, Baum H, Bruckert E, et al. Fasting is not routinely required for determination of a lipid profile: clinical and laboratory implications including flagging at desirable concentration cut-points-a joint consensus statement from the European Atherosclerosis Society and European Federation of Clinical Chemistry and Laboratory Medicine. European heart journal. 2016;37(25):1944-58.
9. Øyri LKL, Bogsrud MP, Kristiansen AL, Myhre JB, Astrup H, Retterstøl K, et al. Cholesterol at ages 6, 12 and 24 months: Tracking and associations with diet and maternal cholesterol in the Infant Cholesterol Study. Atherosclerosis. 2021;326:11-6.
10. Oyri LKL, Bogsrud MP, Kristiansen AL, Myhre JB, Retterstol K, Brekke HK, et al. Infant cholesterol and glycated haemoglobin concentrations vary widely-Associations with breastfeeding, infant diet and maternal biomarkers. Acta Paediatr. 2020;109(1):115-21.
11. Congiu M, Reali A, Deidda F, Dessì A, Bardanzellu F, Fanos V. Breast Milk for Preterm Multiples: More Proteins, Less Lactose. Twin Res Hum Genet. 2019;22(4):265-71.
12. Thomsen LCV, Daltveit AK, Krogvik KT, Næss TP, Klungsøyr K, Akerkar RR. Annual report from the Medical Birth Registry of Norway 2023. Pregnancy and births in Norway. Norwegian Institute of Public Health; 2023.

13. Roman A, Ramirez A, Fox NS. Prevention of preterm birth in twin pregnancies. Am J Obstet Gynecol MFM. 2022;4(2s):100551.

VERSION 2 - REVIEW

**Reviewer**	**3**
**Name**	**Wallace, McKenzie**
**Affiliation**	**The Ohio State University**
**Date**	**10-Mar-2025**
**COI**	

The authors have addressed my concerns and I have no further comments
